# Sacroiliitis secondary to catheter-related bacteremia due to *Mycobacterium abscessus (sensu stricto)*

**DOI:** 10.1186/1476-0711-13-9

**Published:** 2014-01-30

**Authors:** Chrislène Laurens, Geneviève Héry-Arnaud, Raphael Chiron, Eric Oziol, Hélène Jean-Pierre, Nicolas Bouzinbi, Philippe Vande Perre, Anne-Laure Bañuls, Sylvain Godreuil

**Affiliations:** 1 Centre Hospitalier Régional Universitaire (CHRU) de Montpellier, Département de Bactériologie-Virologie, Montpellier, France; 2 Centre Hospitalier Régional Universitaire (CHRU) de Brest, Unité de Bactériologie, Brest, France; 3 Université de Brest, EA3882-LUBEM, SFR148 ScInBioS, Brest, France; 4 Centre Hospitalier Régional Universitaire (CHRU) de Montpellier, Centre de Ressources et de Compétences pour la Mucoviscidose, Montpellier, France; 5 Centre Hospitalier de Beziers, Beziers, France; 6 Université Montpellier 1, Montpellier, France; 7 UMR 5119 (UM2, CNRS, IRD, IFREMER, UM1), Equipe Pathogènes et Environnements, U.F.R. Pharmacie, Montpellier, France; 8 INSERM U 1058, Infection by HIV and by agents with mucocutaneous tropism: from pathogenesis to prevention, Montpellier, France; 9 MIVEGEC, UMR IRD 224-CNRS 5290, Montpellier Universités 1 et 2, Montpellier, France

**Keywords:** Sacroiliitis, Catheter-related, Bacteremia, *Mycobacterium abscessus* complex, MultiLocus sequence typing method, Variable-number tandem-repeat method

## Abstract

We describe a case of sacroiliitis secondary to catheter-related bacteremia due to *Mycobacterium abscessus (sensu stricto)*. This case confirms that MultiLocus sequence typing and variable-number tandem-repeat methods are very robust techniques to identify the pathogen species and to validate molecular epidemiological links among complex *M. abscessus* isolates.

## Background

*Mycobacterium abscessus* species are rapidly growing mycobacteria (RGM) that belong to the group of non-tuberculous mycobacteria. Many RGM species have been identified and *M. abscessus*, *M. chelonae* and *M. fortuitum* are the most important species that cause human diseases. The *M. abscessus* complex (i.e., *M. abscessus sensu lato*) is the most pathogenic and chemotherapy-resistant RGM [1] and is divided in three species (*M. abscessus sensu stricto*, *M. massiliense* and *M. bolletti*) [2] that are consistent with the MLST clustering. More recently, Leao et al. [3] have proposed to group together *M. bolletii* and *M. massiliense* and reclassify them as *M. abscessus subsp. bolletii*. Here, we present a case of sacroiliitis secondary to catheter-related bacteremia due to *Mycobacterium abscessus (sensu stricto).*

## Case report

In January 2007, a 37-year/old woman with fever and infected jugular catheter was admitted to the Emergency Department of the Montpellier University Hospital. She had a past history of Münchausen syndrome, a psychiatric factitious disorder. The patient admitted to injecting herself with tap water in his jugular catheter.

The jugular catheter was inserted one month earlier for empiric antibiotic administration (ceftriaxone and trimethoprim-sulfamethoxazole) at home to treat a radiologically suspected sacroiliitis with negative microbiological investigations of the bone biopsy. Indeed, bone biopsy specimen culture in Columbia sheep blood agar, Chocolate agar, MacConkey agar (BioMérieux, Lyon, France) and Schaedler broth (Becton, Dickinson) as well as the specific research of mycobacterial infection using the BacT/ALERT®3D automated culture system (BioMérieux, Lyon, France) and solid Löwenstein-Jensen (LJ) medium remained negative after 10 days and 12 weeks, respectively. On the basis of clinical and radiological signs, non-infectious sacroiliitis was excluded. After removal, the jugular catheter was cultured on blood agar according to the Brun-Buisson quantitative broth dilution culture technique [4]. Two sets of peripheral blood cultures were established in BacT/ALERT aerobic and anaerobic resin bottles (BioMérieux, Lyon, France). After incubation at 37°C using the BacT/ALERT®3D system (BioMérieux, Lyon, France) for two days, the two aerobic blood cultures became positive for a pleomorphic Gram-positive bacillus. Subcultures of the two aerobic blood cultures and of the catheter culture were performed on Columbia sheep blood agar and after three additional days of incubation, the growth of an irregular Gram-positive, rod-shaped and strictly aerobic organism was observed in all three. Catalase production was detected. Microscopic analysis of the three subcultures after Ziehl-Neelsen (ZN) staining revealed the presence of acid-fast bacilli (AFB) consistent with mycobacteria. After subculture on Middlebrook medium, growth of rough, non-pigmented colonies was noted within five days. The commercial GenoType Mycobacterium CM multiplex line probe assay (Hain Lifescience, Nehren, Germany) identified the organism as *Mycobacterium abscessus*. As the patient did not present symptoms, pelvic radiograph did not detect radiological progression of sacroiliitis and fever disappeared 48 hours after catheter removal, no additional microbiological examination (e.g., sets of peripheral blood cultures) has been made and antimicrobial therapy was postponed. Into the post-hospitalization period, the lack of compliance in this patient did not allow regular medical monitoring. Eight months later, the patient was hospitalized again for severe progression of sacroiliitis (confirmed by computed tomography scan imaging) associated with fistulization to the skin and fever. Fluid from the fistula was collected by fine-needle aspiration for bacteriological investigations. Direct microscopic examination after ZN staining showed the presence of AFB and the mycobacterial infection was confirmed by culture. By using the same routine molecular investigations as before, the fistula isolate was identified as *M. abscessus*. Oral anti-mycobacterial therapy (clarithromycin + ethambutol + ciprofloxacin) was started and continued for a total period of eight months followed by one month treatment with clarithromycin alone. The minimum inhibitory concentrations (MICs) of different antimicrobial drugs on the four isolates was determined using broth microdilution panels (Sensititre RAPMYCO; Trek Diagnosis Systems, Cleveland, Ohio, USA) [5]. The method and guidelines for the interpretation of results were those of the Clinical and Laboratory Standards Institute [6]. The test results were identical for the four isolates and confirmed resistance to doxycycline (MIC ≥ 16 μg/ml), trimethoprim-sulfamethoxazole (MIC: 8/152 μg/ml) and susceptibility to amikacin (MIC: 2 μg/ml), cefoxitin (MIC: 8 μg/ml), ciprofloxacin (MIC: 1 μg/ml), clarithromycin (MIC: 0.125 μg/ml), imipenem (MIC: 2 μg/ml), linezolid (MIC: 2 μg/ml) and intermediate susceptibility to moxifloxacin (MIC: 2 μg/ml). For clarithromycin, the same MIC value was obtained after 5 days and 14 days of incubation. Six months after the end of the antibiotic therapy, the patient was well without fever or clinical and radiological signs of disease progression.

**Figure 1:**
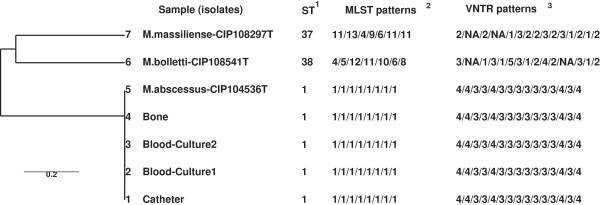
**Tree derived by using the unweighted pair group method with average linkages (UPGMA) based on combined data, MLST**^**2 **^**patterns (including those from the analysis of *argH*, *cya*, *glpK*, *gnd*, *murC*, *pta *and *purH*) and VNTR**^**3 **^**profiles (including those from the analysis of 3416, 4356, 3163, 4038, 4093, 3320, 2177, 3398, 2220, TR45, TR109, TR116, TR150, TR155, TR172 and NA = not available) (7, 22) of the four human isolates and three reference strains.** Sequence types (ST)^1^.

The four clinical isolates (two from blood, one from the catheter and one from the sacroiliac fistula) were then characterized by using the Multilocus Sequence Typing method (MLST), based on seven housekeeping genes (*arg*H, *cya*, *glp*K, *gnd*, *mur*C, *pta* and *pur*H) [1]. Allele and sequence type (ST) queries were performed directly using the *M. abscessus* MLST database (http://www.pasteur.fr/recherche/genopole/PF8/mlst/Myco-abscessus.html). A phylogenetic tree was generated, based on the nucleotide sequences of the supergene obtained by concatenating the seven loci for the four clinical isolates and three reference strains (*M. abscessus* CIP 104536 T, *M. massiliense* CIP 108297 T, *M. bolletii* CIP 108541 T), using the MEGA software, version 5.2 (http://www.megasoftware.net/) (data not shown). The four mycobacterial isolates were assigned to the type strain of *M. abscessus* (*sensu stricto)*. To assess the clonal links, the four isolates were genotyped by using the variable-number tandem-repeat (VNTR) method (with a 15-locus set) associated with MLST analysis (Figure 1) [7,8]. The four isolates had the same sequence type (ST1, which corresponds to the *M. abscessus* (*sensu stricto)* type strain) and the same VNTR profile, based on the analysis of the 15 loci (Figure 1).

## Discussion

*M. abscessus* is an ubiquitous organism found in soil and water, worldwide [9]. The most common clinical manifestation of *M. abscessus* infection is probably chronic lung infection, usually in elderly women with bronchiectasis or in young adults with cystic fibrosis [10]. *M. abscessus* infections are commonly localized to skin and soft tissues, especially after trauma or surgery. Disseminated infections are infrequent and occur especially in immunocompromised hosts [11]. Su et al. [12] described a patient in whom catheter-related *M. abscessus* bacteremia was confirmed by positive blood cultures. *M. abscessus* infections have also been associated with injection of contaminated normal saline and drugs [12]. Usually, the entry route in the organism is known. In our case, the patient (who has a factitious disorder) injected tap water in her central venous catheter. Some studies have reported the presence of *M. abscessus* in tap water, and its ability to form biofilms and to resist to chlorine contributes to its survival and colonization of the water distribution systems [13,14]. Although no investigation was performed to confirm the presence of the same organism in the patient’s environment, the mycobacterial isolate could have been introduced in the blood circulation by contamination of the catheter through the injection of tap water. However, a secondary bone infection or osteomyelitis due to *M. abscessus* bacteremia is infrequent and, classically, *M. abscessus* bacteremia has an indolent course in patients and animal models [12]. In our case, the unusual severity of the disease (sacroiliitis) could have been caused by different factors, such as (i) the presence of a pre-existing sacroiliac joint lesion that could have facilitated the *M. abscessus* infection; (ii) the infection by a rough strain of *M. abscessus* with more virulence factors than smooth strains [15-17]; (iii) the lack of treatment after the identification of *M. abscessus* species in the catheter and blood specimens.

The major threat posed by *M. abscessus* is its antibiotic resistance. This species is probably the most resistant species among pathogenic RGMs [9,18] and its antimicrobial treatment remains a challenge. Therapy duration and the antimicrobial regimens for catheter-related *M. abscessus* bacteremia complicated by osteomyelitis are not clearly defined because of the lack of controlled trials to determine the optimal treatment [19,20]. Combination therapy with at least two effective parenteral agents (amikacin plus cefoxitin, or imipenem), followed by oral clarithromycin monotherapy for weeks to months, has been suggested [18]. Moreover, in the case of catheter-related RGM bacteremia, removal of the catheter is essential because of the high rate of uncontrolled or relapsing bacteremia, even after prolonged antimicrobial therapy. In the present case, despite inappropriate antimycobacterial treatment (an initial parenteral antimycobacterial treatment with amikacin and cefoxitin, or imipenem was not carried out), the patient did not present clinical and radiological evidence of sacroiliitis relapse.

The diagnosis of disseminated RGM infection secondary to catheter-related RGM bacteremia presented several difficulties. Indeed, the definition of catheter-related bacteremia must follow specific criteria: presence of clinical features of bloodstream infection; growth of the same microorganism in the peripheral blood and the catheter; and absence of other apparent sources of infection [21]. Moreover, the interval between the diagnosis of catheter infection, catheter-related bacteremia (positive blood culture) and positive deep infection site (visceral, osteo-articular) culture was of several months. The combination of VNTR and MLST-based DNA fingerprinting is a practical discriminatory procedure to confirm epidemiological molecular links among different *M. abscessus* isolates. Indeed, these molecular tools have been successfully used to explore the genetic links among intra- or inter-patients’ *M. abscessus* isolates, such as in a cohort of pediatric patients with cystic fibrosis [7,8,22,23]. In our case, the genetic data strongly suggest that the sacroiliitis secondary to catheter-related bacteremia was due to the same *M. abscessus (sensu stricto)* isolate from the infected jugular catheter.

Although the differentiation of *M. abscessus*, *M. massiliense* and *M. bolletii* does not comply with the latest nomenclature recommendations [3], it still retains some sense concerning, for instance, the differences in macrolides resistance [24] or in disease phenotype and progression [23]. MLST (targeting 7 housekeeping genes) and multi-spacer sequence analysis are considered very robust methods for identifying species within the *M. abscessus* complex [1]. However, these techniques require genomic sequencing that is relatively costly and time consuming. Recently, Shallom et al. described a simple and not expensive Polymerase Chain Reaction (PCR)-based method to differentiate *M. abscessus* from *M. massiliense* and *M. bolletii* and to subtype *M. abscessus* and *M. massiliense* isolates [25]. Moreover, matrix-assisted laser desorption ionization–time of flight mass spectrometry (MALDI-TOF MS) [26] was recently used to efficiently differentiate the species of the *M. abscessus* complex. This technology appears to be a good alternative to the MLST method.

## Conclusion

In conclusion, we reported a documented case of sacroiliitis secondary to catheter-related bacteremia due to *Mycobacterium abscessus (sensu stricto)*. RGM are infrequently involved in catheter-related infections complicated by disseminated infections. Molecular identification methods and drug susceptibility testing are essential for rapid diagnosis and for prompt and adequate antimicrobial therapy.

## Consent

Written informed consent was obtained from the patient for the publication of this Case report and any accompanying images. A copy of the written consent is available for review by the Editor-in-Chief of this journal.

## Competing interests

The author declares that I have no competing interests and any accompanying images.

## Authors' contributions

CL, GHA, NB, SG ideated this case report and did most writing, supported by PVDP and ALB. RC and EO, HJP have been involved in drafting the manuscript and have made substantial contributions to acquisition of data. All authors read and approved the final manuscript.
